# The role of the Clubhouse model in addressing health inequalities in rural Wyoming: a qualitative study

**DOI:** 10.1080/17482631.2026.2743991

**Published:** 2026-10-08

**Authors:** Ruth Korankye, Gloria Oladeji, Lauren Gilbert

**Affiliations:** a Division of Kinesiology and Health Promotion, University of Wyoming, Laramie, WY, USA; b Department of Human Development and Family Sciences, University of Connecticut, Storrs, CT, USA; c Rural Health Institute, University of Wyoming, Laramie, WY, USA

**Keywords:** Clubhouse model, mental health, rural health, health inequities, social determinants of health, qualitative study

## Abstract

**Objectives:**

To examine health inequalities faced by individuals living with mental illness in rural Wyoming and to explore how the Clubhouse model helps address these challenges.

**Methods:**

This qualitative secondary analysis used semi-structured interview data from a prior study on mental health stigma in rural Wyoming. Sixteen current Clubhouse members (*n* = 12) and staff (*n* = 4 ) were recruited from the only Clubhouse in Wyoming between August and September 2024. Data were analyzed using Braun and Clarke's reflexive thematic analysis in NVivo.

**Results:**

Two major themes emerged: contributors to health inequalities and the Clubhouse’s efforts to address these inequalities. Contributors included limited access to mental health services and structural barriers to essential services, which negatively affected participants’ mental health. Clubhouse efforts addressed multiple social determinants of health, including economic stability, health care access and quality, neighborhood and built environment, and social and community context.

**Conclusions:**

Individuals living with mental illness in rural communities experience persistent health inequalities that negatively affect their mental health. Participants described the Clubhouse model as a supportive environment that can help address these challenges and promote recovery.

Approximately 1 in 5 individuals in the United States reside in rural areas, where residents experience a higher risk of mortality than those in urban areas due to demographic, environmental, economic, and social factors (CDC, 2025). An estimated 6.5 million rural residents live with a mental illness (Morales et al., 2020). Rural suicide rates have exceeded those in urban areas for over two decades, with suicide remaining a leading cause of death nationwide (CDC, 2025). Nearly half of suicide deaths involve a documented mental health diagnosis, and up to 90% of individuals who die by suicide show symptoms of a mental health disorder based on information from relatives and psychological autopsies (Brådvik, 2018; National Alliance on Mental Illness, 2023).

Although the prevalence of mental health disorders is similar in rural and urban communities, rural residents face greater barriers to addressing their mental health needs (Mack et al., 2022; Morales et al., 2020). These disparities are largely driven by shortages and uneven distribution of mental health providers, limiting access to care and contributing to worsening conditions (Andrilla et al., 2018; Kepley & Streeter, 2018; Mack et al., 2022).

Mental health disorders are shaped not only by biological factors but also by social determinants of health (SDOH), including economic stability, education, health care access and quality, neighborhood and built environment, and social and community context, which influence both the prevention and treatment of mental health conditions (Compton & Shim, 2015; U.S. Department of Health & Human Services, 2025).

The Clubhouse model is a community–based service designed to support and empower individuals with mental disorders by fostering self-esteem and providing opportunities for employment, socialization, education, and housing (Clubhouse International, 2025). Previous research highlights how Clubhouse participation improves employment outcomes by equipping members with workforce skills, knowledge, and social support (Bouvet et al., 2021; Chen et al., 2020; Fekete et al., 2021). The Clubhouse has also been described as a positive environment that enhances relationship building, coping strategies, and a shift in focus from their condition to their abilities (Carolan et al., 2011; Chen, 2017).

However, most Clubhouse research has focused on only one or two SDOH domains, particularly economic stability and social and community context (Kinney et al., 2022), with limited attention to broader SDOH impacts in rural settings. Approximately 122 million people in the United States reside in a Mental Health Professional Shortage Area (Mental HPSA), with rural areas disproportionately affected (National Center for Health Workforce Analysis, 2024). Wyoming is among the most rural states in the nation, with approximately 561,187 residents living in areas with limited access to mental health professionals, despite an estimated 88,000 individuals experiencing mental health conditions (National Alliance on Mental Illness, 2023; Scott & Koller, 2019). Notably, the state has only one Clubhouse serving 23 counties, and no studies have examined its role in addressing the social determinants of health in Wyoming.

This study was guided by a recovery-oriented framework grounded in the SAMHSA definition of recovery. Recovery is a person-centered, non-linear process focused on improving well-being and supporting individuals to reach their potential at their own pace (SAMHSA, 2025). It emphasizes hope, empowerment, self-direction, and meaningful living rather than solely symptom reduction (Davidson et al., 2005). This framework aligns with the Clubhouse model, which promotes member autonomy, collaboration, and engagement in purposeful activities within a supportive community environment (Clubhouse International, 2025). It informed the study’s focus on how social and structural support within the Clubhouse contributes to recovery, empowerment, and social connection in rural settings.

This study explored the self-reported health challenges experienced by individuals living with mental illness in Wyoming and how the Clubhouse model addresses these challenges, using semi-structured qualitative interviews. The study addressed the following research questions: (1) How do Clubhouse members describe their experiences of promoting mental health wellness in rural Wyoming? (2) What are the perceptions of Clubhouse members and staff regarding the Clubhouse model’s role in addressing mental health inequities in Wyoming?

## Methods

### Study design

This study employed a qualitative secondary analysis design, in which existing interview data from a previously published study were re-analyzed to address new research questions focused on health inequities and social determinants of health (Heaton, 2004; Ruggiano & Perry, 2019). The original study examined mental health stigma and Clubhouse responses in rural Wyoming (Korankye et al., 2025). Secondary analysis was appropriate because the original semi-structured interviews generated rich narrative accounts of participants' broader lived experiences, including health challenges and structural barriers that extended well beyond the primary study's focus on stigma. This design enabled an in-depth examination of how individuals living with mental illness experience health inequalities and how the Clubhouse model addresses these challenges.

### Study settings and participants

Data for this secondary analysis were drawn from a prior study conducted in collaboration with the only Clubhouse in Wyoming, serving individuals with mental illness across the entire state. The study used all 16 semi-structured interviews (12 Clubhouse members and 4 staff) from the original research (Korankye et al., 2025). The researchers did not have previously established relationships with participants prior to interviews. A summary of the characteristics of Clubhouse members and staff is presented in Tables 1 and 2, respectively. Participants were purposively sampled with Clubhouse staff support via flyers between August and September 2024; full recruitment and eligibility details have been previously reported (Korankye et al., 2025). This sampling strategy was limited to current members and staff who were actively involved in Clubhouse activities. This process may have excluded individuals who had discontinued participation or faced barriers to accessing the Clubhouse due to unavailability, potentially introducing selection bias toward more favorable perceptions of the Clubhouse.

**Table 1.** Demographic characteristics of Clubhouse members (*n* = 12).

Characteristic	*n* (%)
**Age**	
20–30 years	2 (16.7)
31–40 years	2 (16.7)
41–50 years	2 (16.7)
51–60 years	4 (33.3)
61–70 years	1 (8.3)
71–80 years	1(8.3)
**Gender**	
Male	4 (33.3)
Female	7 (58.3)
Other	1 (8.3)
**Race/Ethnicity**	
White	11 (91.7)
Native Hawaiian or Pacific Islander	1 (8.3)
**Duration of membership**	
Less than 6 months	5 (45.5)
Between 6 months and 5 years	5 (45.5)
More than 5 years	1 (9.1)
**Employment Status**	
Employed	3 (25)
Not Employed	9 (75)
**Mental Health Diagnosis**	
ADHD	3 (25.0)
Anxiety	5 (41.7)
Bipolar disorder	5 (41.7)
Borderline personality disorder	2 (16.7)
Depression	3 (25.0)
Dissociative amnesia	1 (8.3)
Psychosis	1 (8.3)
PTSD	6 (50.0)
Schizophrenia	5 (41.7)

Note: All percentages are based on the total number of members (*n* = 12), except for duration of membership (*n* = 11), as one participant did not report this information. Some participants had multiple diagnoses; therefore, percentages for mental health diagnoses do not add up to 100%.

**Table 2.** Demographic characteristics of Clubhouse staff (*n* = 4).

Characteristic	*n* (%)
**Age**	
31–40 years	1 (25)
51–60 years	3 (75)
**Gender**	
Male	0 (0)
Female	4 (100)
**Race/Ethnicity**	
White	4 (100)
**Duration of Employment**	
Less than 6 months	1 (25)
Between 6 months and 5 years	3 (75)

### Ethical considerations

The study received approval from the University of Wyoming Institutional Review Board (IRB Number: IRB-2024-228), in accordance with the principles of the Declaration of Helsinki. All participants provided written informed consent online prior to participation, including the option to withdraw voluntarily. Pseudonyms were used to ensure confidentiality (Korankye et al., 2025). As part of the informed consent process, participants were told the overarching goals of the research: (1) to understand the individual, community, and sociocultural perceptions of the Clubhouse model of recovery support in rural communities and (2) to investigate how the Iris Clubhouse supports and contributes to the long-term recovery of rural individuals.

### Data collection

Detailed data collection procedures have been previously described (Korankye et al., 2025). Semi-structured interviews were co-developed with the Clubhouse director and conducted in August–September 2024, based on participants' preference for in-person or Zoom. Clubhouse members were asked about health disparities experienced while managing mental conditions and Clubhouse support strategies; staff interviews focused on their perceptions of member inequities and Clubhouse responses (Korankye et al., 2025). Interviews were conducted by LG, a college professor with a PhD in Sociology and extensive training and experience in data collection over the last 11 years. Interviews lasted 15–75 minutes (depending on participant availability and membership duration), were audio-recorded, professionally transcribed (in person) or Zoom-transcribed, and verified by research assistants. Researchers also recorded field notes during and after the interviews, which helped to shape the preliminary analysis. Participants received $100 gift cards as agreed-upon incentives with the Clubhouse director (Korankye et al., 2025).

### Data analysis

The secondary data were analyzed using Braun and Clarke's reflexive thematic analysis in NVivo, a qualitative analysis software (Braun & Clarke, 2021). Two researchers independently coded all 16 transcripts, bringing different perspectives to the data. These perspectives were compared through team discussions to enhance analytical insights and refine emerging themes. This iterative and reflective process was overseen by the study's supervisor to ensure the themes were grounded in the data and aligned with the study's objectives. Detailed analysis procedures, research positionality, and trustworthiness are reported in (Korankye et al., 2025).

The research team's racial backgrounds (African American, Ghanaian, Nigerian) and academic backgrounds in sociology, public health, and mental health could influence the analysis process. Therefore, each member was asked to reflect on their background and consider how these perspectives may shape data interpretation. Several meetings were held to discuss and compare individual perspectives. The study incorporated researcher triangulation, persistent observation, and reflexivity to ensure its trustworthiness (Korankye et al., 2025).

## Results

The data analysis identified two main themes: contributors to health inequalities experienced by individuals living with mental illness in rural Wyoming and the Clubhouse’s efforts in addressing them. A thematic table summarizing themes, subthemes, illustrative quotes, participant groups, and SDOH domains is presented in Table 3. The findings also include a thematic map illustrating the relationships among the structural barriers participants experience, the Clubhouse activities that help address these barriers, the recovery processes that could emerge from these barriers, and the perceived outcomes reported by participants (Figure 1).

**Table 3.** Themes, subthemes, illustrative quotes, participant groups, and SDOH domains.

Theme	Subtheme	Illustrative Quotes	Participants	SDOH Domain
**Theme 1: Contributors to health inequalities**	Limited access to mental health services	“It's really hard to get mental help when you don't have insurance in the State of Wyoming”	Member	Health care access and quality
	Structural barriers to essential services	“This bus system is the worst bus system I have ever seen in my entire life...”	Member	Neighborhood and built environment
**Theme 2: Clubhouse's efforts to address health inequalities**	Supporting members toward economic stability	“We have our transitional employment, that's a 6-month program...helping them get back into feeling comfortable working...”	Staff	Economic stability
	Promoting housing stability	“When I first started there, I was homeless; I currently have an apartment”	Member	Neighborhood and built environment
	Facilitating access to mental health services	“At least it is giving some of these people that don't have insurance a place to go...get them set up with a case manager”	Member	Health care access and quality
	Fostering community and member advocacy	“The Clubhouse is a community...building those friendships and connections... to help them feel like somebody.”	Staff	Social and community context

Note: SDOH = Social Determinants of Health; Member = Clubhouse member; Staff = Clubhouse staff member.

**Figure 1.**
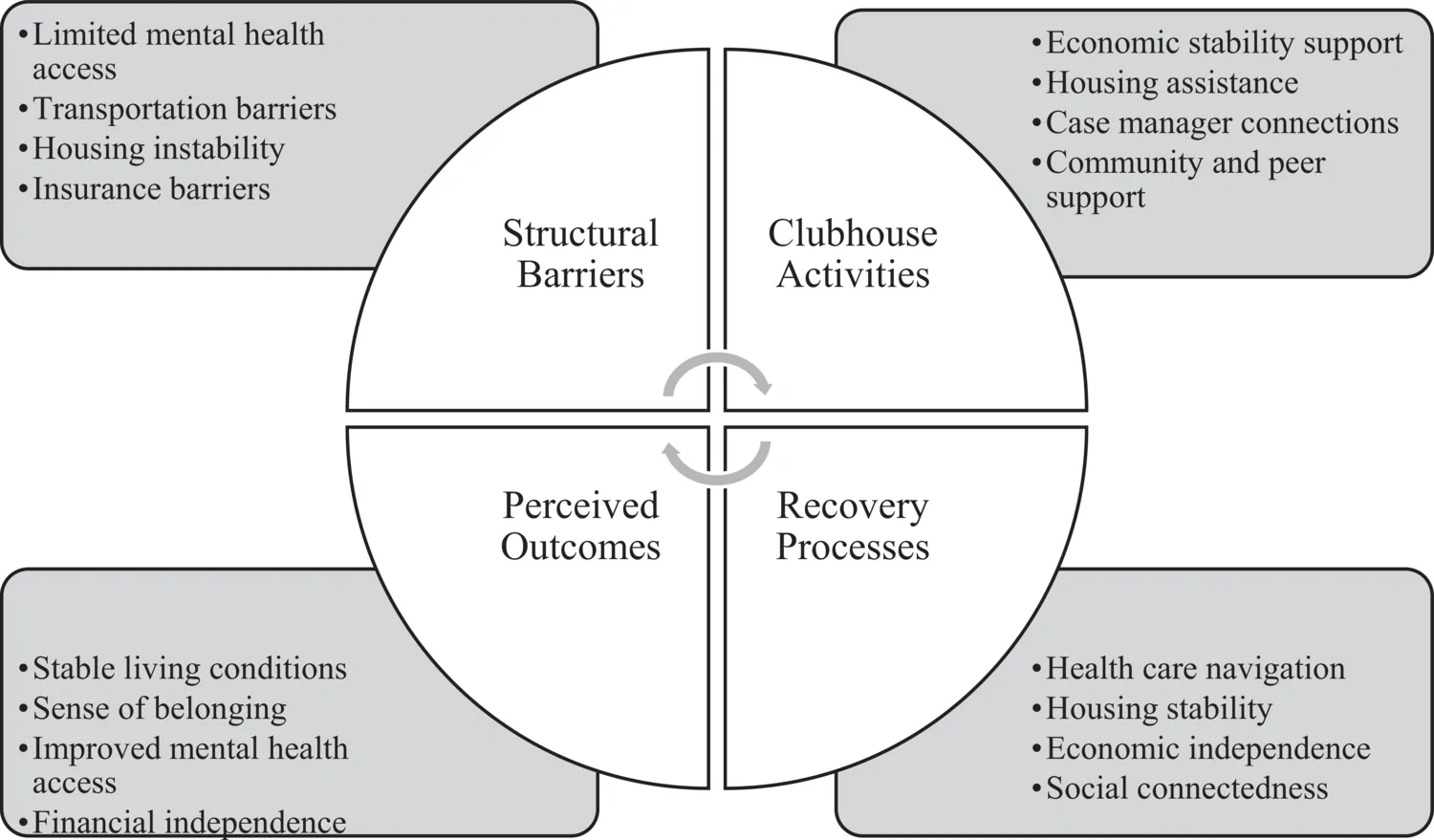
Thematic map of the Clubhouse model's role in addressing health inequalities in rural Wyoming. ***Note.*** Recovery processes are aligned with SAMHSA's four dimensions of recovery: health, home, purpose, and community.

### Theme 1: contributors to health inequalities

Limited access to mental health services and structural barriers to essential services were reported by participants as key contributors to health inequalities in rural Wyoming.

#### Subtheme 1: limited access to mental health services

Many participants highlighted challenges in accessing mental health services in their communities, with some recounting longer wait times to see a therapist or psychiatrist. Cheryl, a Clubhouse member, stated, “Not easy. I waited for this new doctor for months and months. It was hard to get in to see this new doctor. It's hard to get in to see a therapist.” Angie, a Clubhouse member, added: “There were no psychiatrists who were doing private practice. Would you believe that in a place like this? Where you didn’t have private doctors that you had to go to a center.” These experiences reflect the burden faced by individuals living with mental illness due to the shortage of mental health practitioners in rural communities, underscoring how systemic gaps in rural mental health infrastructure significantly shape these individuals' lives.

Another contributing factor to accessing mental health care was a lack of insurance. Emily, a Clubhouse member, noted: “It wasn't easy until I got SSI and got Medicaid, and then it was easy to find treatment because I had insurance to get the medicine and see the doctors. It's really hard to get mental help when you don't have insurance in the State of Wyoming.” This illustrates how insurance can serve as a gateway to mental health care in rural Wyoming, where its absence creates significant barriers to accessing services for individuals living with mental illness.

#### Subtheme 2: structural barriers to essential services

Structural barriers, such as limited access to housing and transportation, were identified as major concerns for participants. Participants had varying experiences with transportation; those with personal vehicles or transportation assistance did not experience these barriers. Emily, a Clubhouse member, emphasized:

*It's fairly easy. I have a car. My friend has a car, luckily, I have SSI, so I can put money back for gas and stuff, so my transportation is a lot easier than some other people's that have to rely on the bus system, Uber, or taxis.*

However, those without personal transportation reported significant challenges. Cheryl, a Clubhouse member, noted: “Well, not easy, because this bus system is like in the mornings, it runs every hour, after that, it's every 2 hours. This bus system is the worst bus system I have ever seen in my entire life...” Libby, a Clubhouse member, also described her experience:

*I used to go to the clinic. Do you know where the counseling center is? I used to go to them, and they're all the way up on the hill, and I take the bus everywhere, so it would take me like an hour and a half to get there, and an hour and a half to get home. And I would spend 2 hours there a day, to get my appointments once a month, and it's just ridiculous.*

These findings suggest an inequality in accessing timely mental health care, disproportionately affecting those without personal vehicles, further compounding the challenges already faced by individuals living with mental illness in rural communities.

Housing instability was another challenge for individuals with mental illness, particularly for those with a felony record or those still in recovery. Abby, a Clubhouse staff member, explained:

*Once you had a felony drug conviction 10 years ago, sorry, you don't qualify. Well, that was 10 years ago. So, you don't qualify - just like things like that, once they're evicted from the housing here, they can't use any of the [local] housing programs, period.*

Sandra, a Clubhouse staff member, added:

*We've had some members being kicked out of where they're living, because maybe they're yelling, or just part of that mental illness. But there's also housing; we have the housing authority, which is supposed to help people get housing. But if you're a felony or you've been kicked out of an apartment, then where do they go next, and so, just trying to find that next level of housing can be very complicated.*

These narratives reflect the structural exclusion faced by these individuals, where past records and behavioral symptoms could perpetuate housing instability.

### Theme 2: the Clubhouse’s efforts to address health inequalities

The Clubhouse was described as a community-based support system that addresses multiple social determinants of health, including supporting members' economic stability, promoting housing stability, facilitating access to mental health services, and fostering a sense of community and member advocacy.

#### Subtheme 1: supporting members toward economic stability

Several staff participants highlighted that the Clubhouse supports members’ economic stability through its transitional employment program.

Sandra, a staff member, explained:

*We have our transitional employment, that's a 6-month program where we're helping them. It's a connection through the employer where we have the 2 positions. We put our members in there for 6 months, and it's helping them get back into feeling comfortable working or seeing if they can work, just getting their skills to put on a resume or an application, and then it's a building block to next, where we still can advocate for them.*

Beyond the transitional employment program, Tiffany, a staff member, illustrated how the Clubhouse contributes differently to economic stability:

*Helping people to find jobs and keep jobs, that's an obvious way that we help people with economic stability…there are other things that go into economic stability, such as you have to have all of those lower-level needs met, such as housing, food, water...we can help in those ways by making sure that people have access to federal programs, whether it's SNAP benefits...building relationships, having healthy relationships, all of those things play into your ability to keep a job and have a job.*

These perspectives reveal that the Clubhouse not only prepares members for employment through structured programs but also recognizes economic stability as a multidimensional concept, reflecting a holistic approach to supporting members' financial independence.

The Clubhouse was perceived as providing accessible resources that facilitated members’ job search. Angie, a Clubhouse member, asserted:

*The Clubhouse has a board that has different job information for us to look at, and I did. I contacted a [local business] through an ad they had on the board. Basically, they have the computers there too, that you could go online. I went on Indeed.*

This reflects how the Clubhouse's resources empower members to take the initiative in their job searches, rather than relying solely on the Clubhouse.

#### Subtheme 2: promoting housing stability

Participants highlighted the Clubhouse's role in addressing housing instability, a common challenge experienced by individuals living with mental illness. Amber, a Clubhouse member, recounted: “They've been really helpful and supportive. When I first started there, I was homeless; I currently have an apartment. I've been in the apartment about a year, a little over a year now.”

Staff members also witnessed these changes. Sandra, a staff member, noted: “We see people that are homeless and don't have jobs, now they have a steady home.” Susan, a Clubhouse member, added: “We keep openings for apartments and post apartments that are taking applications for people in the area. We do support their housing and try and get them…if they're at risk of losing their housing at any time they can come to us.”

These observations suggest that the Clubhouse could serve as a valuable resource for members transitioning from homelessness to stable living conditions.

#### Subtheme 3: facilitating access to mental health services

Participants reported that the Clubhouse helped bridge gaps in accessing mental health services by connecting members to case managers and providing transportation support. Emily, a Clubhouse member, noted:

*There are counselors, there are places, as long as you have insurance, but that's why I'm happy there is the Clubhouse, because at least it is giving some of these people that don't have insurance a place to go, and then getting some form of care and can kind of get them set up with a case manager and work on things.*

This reveals that the Clubhouse can serve as a liaison, connecting individuals disproportionately affected by a lack of insurance to case managers for ongoing support.

Transportation support was emphasized as another pathway through which the Clubhouse facilitated access to mental health care. Cheryl, a Clubhouse member, expressed: “This place has a van that will come pick me up at the Mission and bring me here in the morning. That [counseling center] doesn't even have a van to come and pick you up and take you back...” Stacey, a staff member, added: “Someone picks up people that don't have transportation or don't want to ride the bus.” These narratives suggest that the Clubhouse could help members navigate transportation barriers rural communities often encounter, thereby bridging the gap in formal services.

#### Subtheme 4: fostering a sense of community and member advocacy

Participants characterized the Clubhouse as a community-based recovery environment that supports individuals in their recovery journey. Sandra, a staff member, illustrated:

*The Clubhouse is a community, so the other resources you're going in, you're getting your help, you're leaving. But it's not that community of coming in and being with the people every day, knowing them, and building those friendships and connections. We're more than just a hangout. We're more than just a place to come in and fill out paperwork. We're building those relationships and connections to be able to help them feel like somebody.*

Tiffany, a staff member, added:

*Just having a support network and a place to go, a place to belong, a place to feel needed, wanted and expected...having people who believe in you and believe that recovery is possible. Having a group of people who don't judge and understand because they also are going through similar things.*

Collectively, these findings underscore the importance of a community-based environment where individuals with shared experiences can connect and develop a sense of belonging that extends beyond traditional mental health service delivery.

Members' sense of belonging was further reflected in their pride in Clubhouse membership. Emily, a Clubhouse member, affirmed: “Yeah, I'm proud of the Clubhouse. I am totally proud of somebody knowing.” Stacey, a staff member, echoed this, noting: “They're proud to be members and they have a sense of ownership. This is their clubhouse.”

This sense of pride translated into members publicly disclosing their membership and referring others to the Clubhouse. Scarlet, a Clubhouse member, noted: “To show that I'm struggling with mental health and that it's okay to struggle with mental health.”

Stacey, a staff member, indicated:

*We have what’s called member referrals. So, I’d say over the last month is pretty much when we really started it big. That’s where most of our referrals have come from, someone who’s living with mental illness refers a friend, brings in a friend.*

However, not all members felt comfortable disclosing their membership. Susan, a Clubhouse member, acknowledged: “The majority of them would, but some people would not want to have the stigma stuck on them, especially if they have really haunting memories of prejudices against them.” Together, these experiences highlight that while the Clubhouse fosters a sense of pride and belonging among members, the willingness to publicly disclose membership varied from person to person.

## Discussion

The study’s findings highlight the challenges individuals with mental illness encounter while accessing mental health services and how the Clubhouse model helps address these challenges. Participants emphasized that health inequities may be driven by limited access to mental health services and structural barriers to essential resources, suggesting that multiple domains of the social determinants of health may intersect to shape mental health experiences in rural Wyoming.

### Limited access to mental health services

Limited access to mental health care remains a substantial barrier to improved mental health outcomes in rural areas. Participants' experiences reflected these systemic challenges, including long wait times, limited numbers of mental health professionals, inadequate private facilities, and insurance-related obstacles. In Wyoming, an estimated 96.4% residents live in areas with few mental health providers, contributing to the prolonged wait times to care (Mack et al., 2022). Geographic isolation and low population density in rural communities further limit the availability of private mental health facilities, exacerbating delays in treatment (Rural Health Information Hub, 2025). These delays may contribute to symptom exacerbation and prolonged psychological distress due to unmet mental health needs (França et al., 2023; Negash et al., 2023).

Insurance-related barriers also restrict access to care, particularly for uninsured individuals, consistent with prior research in rural settings (National Center for Health Workforce Analysis, 2024; Rural Health Information Hub, 2025). In 2022, more than half of Wyoming residents who did not seek mental health care cited cost as the barrier (National Alliance on Mental Illness, 2023). This finding underscores the continued impact of cost-related barriers on access to mental health services in rural Wyoming. Strategies, including equitable distribution of mental health professionals, integration of behavioral health into primary care, and improved insurance coverage for mental health services, may reduce wait times and strengthen health care access and quality as social determinants of health (National Center for Health Workforce Analysis, 2024).

### Structural barriers to essential services

Participants described transportation limitations and housing instability as persistent structural barriers to accessing essential services. Limited public transportation options are not unique to Wyoming, but nationally, transportation barriers in rural areas are largely driven by infrastructure limitations, including shortages of vehicles and personnel (Henning-Smith et al., 2017). Transportation barriers have been linked to missed appointments, delayed care, and poorer health outcomes, highlighting how geographic isolation and infrastructure limitations restrict access to both health and social services (Kelly et al., 2016; Wolfe et al., 2020).

Housing instability was identified as a major barrier to recovery, particularly among individuals with felony histories or those experiencing symptom relapses. Relapses during recovery may further increase the risk of eviction, compounding instability. These findings align with SAMHSA’s recovery framework, which identifies a stable home as essential to sustained recovery (SAMHSA, 2025). Unstable housing and homelessness are associated with increased relapse risk, hospital readmission, and poorer health outcomes (Catthoor et al., 2024; Russolillo et al., 2023). Evidence suggests that addressing housing instability can improve mental health outcomes; individuals with stable housing experience fewer emergency department visits than those without housing stability (Srebnik et al., 2013). These findings suggest that housing instability not only reflects structural disadvantages but may also perpetuate cycles of relapse and instability.

## The Clubhouse’s efforts in addressing health inequalities

Participants described the Clubhouse as addressing multiple SDOH domains: economic stability, health care access, housing and neighborhood environment, and social and community context.

### Supporting members toward economic stability

Participants described employment as a major challenge for individuals living with mental illness, particularly when disclosure occurs during job interviews (Charette-Dussault & Corbière, 2019). This challenge may undermine economic stability and worsen mental health outcomes (Junna et al., 2022). The Clubhouses helped mitigate this challenge by supporting workforce reintegration through transitional employment and by providing resources that build skills, confidence, and employability. Consistent with prior research, engagement in Clubhouse programs, especially transitional employment, was associated with improved employment outcomes (Chen et al., 2020; Fekete et al., 2021). By supporting employment, Clubhouses may not only improve members’ financial independence but also contribute to their long-term recovery by fostering purpose and meaning in life (SAMHSA, 2025).

### Promoting housing stability

Participants recounted that the Clubhouse addresses housing instability, a significant barrier to long-term recovery of individuals living with mental illness. The Clubhouse provides essential support to members experiencing homelessness by assisting with apartment searches and helping maintain their homes when challenges arise. The housing support directly addresses the social determinant of health related to the neighborhood and the built environment by providing a safe environment that supports their long-term recovery (National Alliance on Mental Illness, 2022; SAMHSA, 2025; U.S. Department of Health & Human Services, 2025). Given that approximately one in five individuals experiencing homelessness in the United States lives with a serious mental illness, Clubhouse housing support may play a role in addressing the national burden of homelessness (National Alliance on Mental Illness, 2022). However, this effort remains underreported in the existing literature (Catthoor et al., 2024; Russolillo et al., 2023). Expanding the number of Clubhouses, particularly in rural areas, could contribute to addressing housing insecurity and potentially improving the health outcomes of individuals with mental illness (National Alliance on Mental Illness, 2022). However, their effectiveness and feasibility require further evaluation.

### Facilitating access to mental health services

Rural communities experience limited access to mental health services due to shortages of providers, inadequate private facilities, insurance-related challenges, and long wait times (Mack et al., 2022; National Center for Health Workforce Analysis, 2024; Rural Health Information Hub, 2025). These barriers negatively affect health outcomes, contributing to the worsening of symptoms, developing comorbidities, and an increased risk of suicide (Negash et al., 2023). The Clubhouse facilitates connections to case managers and provides support for individuals facing insurance and transportation barriers, thereby supporting continuity of care and addressing SDOH related to healthcare access and quality (U.S. Department of Health & Human Services, 2025). This demonstrates how Clubhouses function as informal linkage systems that help bridge gaps in rural behavioral health service delivery. Increasing the availability of Clubhouses in rural communities could reduce service disparities and support better mental health outcomes.

### Fostering a sense of community and member advocacy

Community, a key dimension of recovery identified by SAMHSA, encompasses relationships and social networks that provide support, friendship, and hope (SAMHSA, 2025). The Clubhouse was described as a supportive community that fosters peer connection through shared lived experiences, offering encouragement and sustaining members throughout their recovery journeys (Raeburn et al., 2016). Unlike some traditional mental health settings, engagement in Clubhouse activities was voluntary, allowing members to pursue recovery at their own pace and promoting autonomy and self-determination (Tanaka et al., 2021). Members also expressed pride in their affiliation with the Clubhouse and actively advocated for its benefit, reflecting the central importance of social connectedness and belonging in recovery-oriented mental health care. However, not all members felt comfortable publicly disclosing their membership, reflecting the persistent stigma surrounding mental illness in rural communities. Together, these findings highlight the Clubhouse as a supportive environment that may foster long-term recovery through peer connection, autonomy, and shared lived experience.

Individuals living with mental illness tend to experience structural barriers at the policy level, such as inadequate insurance coverage, housing discrimination, and mental health provider shortages; however, the Clubhouse addresses these challenges at the individual level by helping members navigate these barriers rather than eliminating them (Compton & Shim, 2015). This individual-level support is essential; nevertheless, broader policy-level interventions are ultimately needed to dismantle the structural inequities that continue to affect this population (Alegría et al., 2023; Compton & Shim, 2015).

## Study limitation

The study had several limitations. The small sample size from Wyoming's only Clubhouse limits the extent to which the perspectives of Clubhouse members and staff can be generalized, as Wyoming's rurality may shape participants' perceptions in ways that differ from those in Clubhouses in other states or settings. Additionally, the study used a secondary analysis, which may not capture all participants' experiences of SDOH, as the original study focused on mental health stigma rather than health inequities.

Recruitment was limited to current members and staff actively involved in Clubhouse activities and was facilitated by Clubhouse leadership, which may have introduced selection bias toward more favorable perceptions of the Clubhouse, as the study excluded perspectives of former members or those who faced barriers to participation. Moreover, the data were self-reported, which may have led participants' accounts to be subject to social desirability bias, potentially influencing the favorability of their responses. Furthermore, variable interview durations, influenced by membership duration, may have yielded less rich data from newer members, potentially limiting the depth of perspectives captured.

The absence of a comparison group also limits the ability to distinguish the Clubhouse's specific contributions to members' well-being from those of non-members. However, the findings provide rich contextual insight into how Clubhouses address social determinants of health in rural communities.

## Study implications

This study advances understanding of how the Clubhouse model addresses key social determinants of health for individuals living with mental illness, highlighting its potential role in supporting recovery and addressing mental health inequities.

Findings have important policy implications, suggesting that expanding Clubhouses, particularly in rural areas, may help reduce persistent social and structural inequities affecting this population. Community-based psychosocial models like Clubhouses may serve as practical approaches to reducing mental health inequities in rural settings and strengthen recovery-oriented systems of care. However, to eliminate these systemic barriers, policy-level interventions, such as redistributing mental health providers, implementing affordable housing policies, and improving rural transportation infrastructure, must be enacted to address the root causes of these structural inequities and ensure health equity for this population.

The results also offer mental health professionals insights to consider referrals to Clubhouses for individuals living with mental illness in rural communities, who could benefit from social support and recovery-oriented resources, thereby increasing awareness of this program among this population.

Future research should examine how Clubhouses influence additional social determinants of health, including health care access, education, and neighborhood environments, using both qualitative and quantitative approaches to assess their broader effects on health equity. Researchers should replicate this study with larger and more geographically diverse populations to capture broader perspectives and further explore how the Clubhouse model addresses health inequities among individuals living with mental illness and help inform evidence-based policy.

## Conclusion

Rural communities continue to experience persistent mental health inequities, including limited access to services and structural barriers to essential resources. This study suggests that the Clubhouse model may play an important role in addressing these challenges by targeting multiple social determinants of health, including economic stability, housing security, access to care, and social connectedness. The findings also highlight that recovery from mental illness in rural settings is shaped not only by clinical care but also by access to stable housing, employment opportunities, and supportive community environments. The Clubhouse model, therefore, represents a holistic, community-based approach that complements traditional mental health services by addressing social and structural factors that influence recovery. Expanding Clubhouse programs in rural areas could be a potential strategy to reduce mental health inequities and strengthen recovery-oriented systems of care.

## Supplementary Material

Supplementary Material Studys_COREQ_Checklist.pdf

## Data Availability

Due to the nature of the research, the qualitative data supporting this study’s findings are not publicly available for ethical reasons related to participant confidentiality and the sensitive nature of the collected information. Participants were assured of confidentiality during the informed consent process, and sharing the data, even in de-identified form, may risk participant identification.
